# Bovine Adenovirus 3-Based Viral Vectors for Veterinary Vaccine Development: Progress, Limitations, and Future Directions

**DOI:** 10.3390/vetsci13090850

**Published:** 2026-08-22

**Authors:** Nattawooti Sthitmatee, Thanya Varinrak, Khwanchai Kreausukon

**Affiliations:** 1 Laboratory of Veterinary Vaccine and Biological Products, Faculty of Veterinary Medicine, Chiang Mai University, Chiang Mai 50100, Thailand; thanya.var@cmu.ac.th (T.V.); khwanchai.kreau@cmu.ac.th (K.K.); 2 Central Laboratory for Teaching and Research, Faculty of Veterinary Medicine, Chiang Mai University, Muang, Chiang Mai 50100, Thailand

**Keywords:** adenoviral vector, BAdV-3, bovine adenovirus, veterinary vaccine, viral vector vaccine

## Abstract

Bovine adenovirus (BAdV)-based vectors, particularly those derived from bovine adenovirus 3 (BAdV-3), are emerging non-human adenoviral platforms for veterinary vaccine development. Adenoviral vectors are attractive for vaccination because they mediate efficient transgene expression, remain largely episomal, and induce robust innate and adaptive immune responses. However, widely used human adenoviral vectors, especially human adenovirus 5 (HAdV-5), may be compromised by pre-existing anti-vector immunity, supporting the development of rare human serotypes and non-human adenoviral alternatives. BAdV-3 is the best-characterized BAdV for recombinant vector engineering and has been used to express heterologous antigens from bovine herpesvirus-1, bovine respiratory syncytial virus, influenza virus, and *Mycobacterium tuberculosis*. Available evidence indicates that BAdV-based vectors can induce humoral, cellular, and mucosal immune responses and support intranasal antigen delivery. Protective efficacy has been demonstrated in selected experimental models, including mice and ferrets for influenza and tuberculosis vaccine candidates, whereas cattle challenge evidence remains more limited and is primarily represented by BHV-1 gD-expressing BAdV-3 vectors. These features make BAdV vectors relevant to bovine respiratory disease control, livestock vaccination, and One Health-oriented vaccine preparedness. Nevertheless, the platform remains at an early developmental stage. Key gaps include limited target-species efficacy data, pre-existing BAdV immunity in cattle, vector shedding, recombination risk, genetic stability, producer cell optimization, manufacturing scalability, and regulatory feasibility.

## Simple Summary

Vaccines are essential for preventing infectious diseases in animals, but many existing vaccines still have limitations, especially for diseases that enter through the nose, lungs, or digestive tract. This review discusses bovine adenovirus, a virus originally associated with cattle, as a possible tool for carrying vaccine instructions into animal cells. This approach may help the body produce protective immune responses without the vaccine virus becoming part of the animal’s genetic material. The aim of this review is to summarize how bovine adenovirus-based vaccine carriers have been developed, how they can be engineered, what immune responses they may stimulate, and how they may be used against important animal diseases. Available studies suggest that these vaccine carriers can deliver different disease targets, stimulate antibody and immune-cell responses, and may be useful for respiratory diseases, combined vaccines, and diseases shared between animals and humans. However, this technology is still developing. More studies are needed to confirm safety, effectiveness in target animal species, large-scale production, and regulatory acceptance. If these challenges are addressed, bovine adenovirus-based vaccine carriers may become useful tools for improving livestock health, reducing disease losses, and supporting public health.

## 1. Introduction

Adenovirus-based vectors are among the most widely used viral platforms for gene delivery and vaccine development. Since adenoviruses were first identified from human adenoid tissues, their biological features have been extensively characterized and later exploited for vector design [1]. These vectors are attractive because they efficiently enter host cells, support high-level transgene expression, have relatively large cloning capacity, infect both dividing and non-dividing cells, and generally remain episomal rather than integrating into the host genome. These properties have supported their use in gene therapy, cancer therapy, immunotherapy, and infectious disease vaccination [2,3,4,5,6].

A major milestone in adenoviral vector development was the establishment of HEK293 cells, which provide E1 functions in trans and allow production of replication-defective adenoviral vectors [7]. Early studies demonstrated adenovirus-mediated in vivo gene transfer, including delivery to respiratory epithelium, establishing the basis for E1-deleted and E1/E3-deleted vectors used in gene therapy and vaccine research [2,5,8,9].

For vaccine development, adenoviral vectors are particularly valuable because they induce strong innate and adaptive immune responses. Intracellular antigen expression enables antigen presentation through MHC class I and class II pathways, promoting antibody responses, CD4^+^ T-cell activation, and cytotoxic CD8^+^ T-cell responses. Thus, immunogenicity that was sometimes a limitation in gene therapy became an advantage for vaccination [3,5,9,10]. The deployment of adenoviral vector vaccines during the SARS-CoV-2 pandemic further demonstrated their utility for rapid antigen design, scalable manufacturing, and emergency vaccine responses [6,10,11,12].

However, human adenovirus (HAdV) vectors, especially HAdV-5 vectors, are limited by pre-existing anti-vector immunity. Neutralizing antibodies and cellular responses against common HAdV can reduce vector entry, antigen expression, vaccine-induced immunity, and repeated homologous boosting [3,9,13,14]. This limitation has driven the development of rare human serotypes, such as Adenovirus serotype 26 (Ad26) and Adenovirus serotype 35 (Ad35), and non-HAdV from chimpanzees, cattle, pigs, dogs, sheep, and birds [9,15,16].

Within this landscape, bovine adenovirus 3 (BAdV-3) has emerged as a promising non-human adenoviral vector for veterinary vaccine development. BAdV-3 is the most extensively studied BAdV for recombinant vector engineering and has been modified to express foreign genes and vaccine antigens. Early work established BAdV-3 as a recombinant expression platform through insertion of a firefly luciferase reporter gene into the E3 region [17]. Subsequent studies constructed E3-deleted BAdV-3 vectors expressing full-length or truncated BHV-1 glycoprotein D (gD) [18], developed replication-defective BAdV-3 expression vectors [19], and evaluated replication-defective and replication-competent gD-expressing BAdV-3 vectors in cattle [20].

BAdV-based vectors have also been evaluated for respiratory pathogens, influenza vaccines, and mucosal immunization. BAdV-3 vectors expressing bovine respiratory syncytial virus (BRSV) and bovine herpesvirus (BHV) antigens support their relevance to bovine respiratory disease vaccine research [21]. BAdV-based influenza vaccines induced immune responses despite pre-existing HAdV immunity and provided protection against H5N1 influenza in mouse models [22,23]. Subsequent studies extended the platform to mucosal tuberculosis vaccination in mice and universal influenza vaccine strategies evaluated in mice and ferrets [24,25].

Nevertheless, BAdV-based vectors remain emerging rather than commercially established platforms. Compared with HAdV-5, Ad26, and chimpanzee adenoviral vectors, BAdV vectors have fewer target-species efficacy data, less manufacturing experience, and limited regulatory familiarity. Key gaps include pre-existing BAdV immunity in cattle, vector shedding, genetic stability, recombination risk, scalable production, and field efficacy [26,27].

A narrative literature search was conducted to identify publications relevant to bovine adenovirus-based vector development and veterinary vaccination. PubMed, Scopus, and Google Scholar were searched using combinations of the terms “bovine adenovirus,” “BAdV,” “adenoviral vector,” “vaccine vector.” The search covered publications from database inception to April 2026. Reference lists of relevant primary studies and review articles were also examined to identify additional publications.

Studies were selected when they provided information directly relevant to biology, molecular characterization, vector engineering, recombinant antigen expression, immunogenicity, protective efficacy, mucosal delivery, biosafety, production, or veterinary vaccine applications. Primary BAdV and particularly BAdV-3 studies were prioritized. Studies of human, chimpanzee, or other animal adenoviral vectors were included when they provided relevant comparative information on vector engineering, pre-existing immunity, manufacturing, safety, or translational development. Publications lacking direct relevance to adenoviral vector biology or vaccine development were excluded. Because this review was designed as a narrative synthesis rather than a systematic review, no formal risk-of-bias assessment or quantitative meta-analysis was performed.

**Figure 1:**
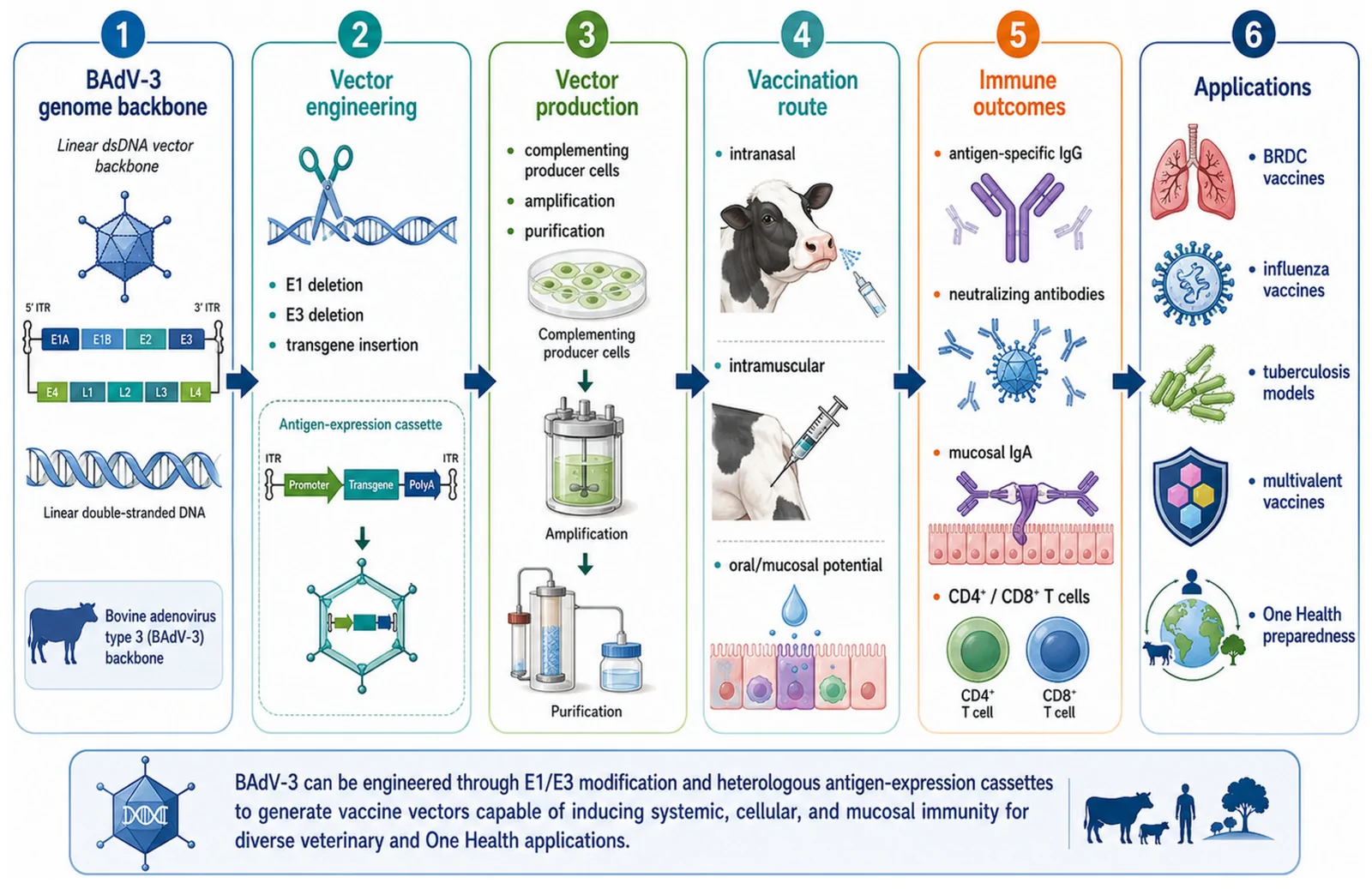
Conceptual overview of BAdV-based viral vector development. BAdV-3 can be engineered through modification of replication-associated or non-essential genomic regions, particularly the early region 1 (E1) and early region 3 (E3), followed by insertion of heterologous antigen-expression cassettes. Replication-defective vectors require complementing producer cells that supply deleted viral functions in trans. Following administration, BAdV-based vaccines can induce systemic antibody, cellular, and mucosal immune responses and have been investigated for respiratory, influenza, intracellular-pathogen, and multivalent vaccine applications. BRDC, bovine respiratory disease complex; IgG, immunoglobulin G; IgA, immunoglobulin A; CD4, cluster of differentiation 4; CD8, cluster of differentiation 8.

This review summarizes the historical development, biological rationale, engineering strategies, vaccine applications, immunological outcomes, advantages, limitations, biosafety considerations, comparative perspectives, and future directions of BAdV-based viral vectors, with emphasis on BAdV-3 as an emerging platform for livestock vaccination, mucosal immunization, multivalent vaccine design, and One Health preparedness. The overall concept of BAdV-based vector development, from vector engineering to vaccine applications, is illustrated in Figure 1.

## 2. Historical Development of Adenovirus-Based Vectors and Vaccines

The development of adenovirus-based vectors represents a transition from basic virology to gene-transfer and vaccine platforms. Adenoviruses were initially studied as infectious agents, but their linear double-stranded DNA genome, efficient cell entry, broad tissue tropism, high-level transgene expression, and strong immunostimulatory capacity later made them attractive for genetic engineering and vaccination [3,4,5,6,28]. Key historical milestones in adenovirus-based vector development and their relevance to BAdV-based vaccine platforms are summarized in Table 1.

### 2.1. Discovery and Early Biological Characterization of Adenoviruses

Adenoviruses were first identified from human adenoid tissue and respiratory disease studies and were later characterized as non-enveloped, icosahedral viruses with linear double-stranded DNA genomes. Their ability to infect epithelial tissues, replicate efficiently, and stimulate host immune responses provided the biological foundation for their later use as vectors [3,5].

Several features made adenoviruses suitable for vector development: stable and genetically manipulable genomes, efficient infection of dividing and non-dividing cells, episomal persistence without routine host-genome integration, and strong innate and adaptive immune activation. These properties supported their early use in gene delivery and later became particularly valuable for vaccine applications [3,6,9,29].

### 2.2. Development of Adenovirus Vectors for Gene Transfer

A key technical milestone was the development of replication-defective adenoviral vectors through deletion of essential viral genes and production in complementing cell lines. HEK293 cells, which provide adenoviral E1 functions in trans, enabled propagation of E1-deleted vectors that could express transgenes in target cells but could not complete productive replication in normal cells [3,7,30,31].

First-generation adenoviral vectors commonly involved E1 deletion for replication deficiency and E3 deletion to increase cloning capacity for foreign expression cassettes. This E1/E3-deleted design became a widely used strategy for therapeutic gene transfer and later vaccine antigen delivery [3,9,31]. The same production principle later influenced non-human adenoviral vectors, including BAdV-based systems, where deleted viral functions are supplied by complementing producer cells during manufacturing [3,7,19].

### 2.3. Transition from Gene Therapy Vectors to Vaccine Vectors

Adenoviral vectors were first developed mainly for gene therapy because they mediate efficient transient transgene expression. However, their strong innate activation, inflammation, vector-specific antibodies, and cellular immune responses limited long-term gene replacement and repeated administration [3,9,30].

In vaccine development, these same immune-stimulating properties became advantages. Adenoviral antigen expression promotes MHC class I presentation and CD8^+^ T-cell responses, while antigen uptake by antigen-presenting cells supports MHC class II presentation, CD4^+^ T-cell activation, and antibody production. Thus, adenoviral vectors can induce both humoral and cellular immunity, making them useful for vaccines against viral infections, intracellular pathogens, and cancer targets [3,4,5,6]. This shift enabled adenoviral vaccines to be explored for diseases such as rabies, HIV, Ebola, malaria, influenza, tuberculosis, and COVID-19 [3,5,10].

### 2.4. Transition to Alternative Adenoviral Vaccine Platforms

The extensive development of HAdV-5 established adenoviruses as effective vaccine-vector platforms but also highlighted limitations associated with pre-existing anti-vector immunity [3,9,13]. This prompted the development of alternative human serotypes, including Ad26 and Ad35, as well as non-human adenoviruses such as chimpanzee adenoviruses (ChAd), and animal adenoviruses [9,10,11,12,13,14,15,16]. These platforms demonstrated that adenoviral backbones from different host species can be engineered for antigen delivery and vaccination. Within this broader development, BAdV-3 emerged as a bovine-origin vector of particular relevance to veterinary vaccinology. Its molecular characterization and subsequent engineering for heterologous gene and vaccine-antigen expression provided the foundation for the BAdV-specific studies discussed in the following sections [17,19,26,27].

## 3. Overview of BAdV

BAdVs are cattle-associated, non-enveloped, icosahedral viruses with linear double-stranded DNA genomes. They are relevant both as bovine pathogens and as viral backbones for recombinant vector development. Among them, BAdV-3 has been the most extensively characterized and is the principal BAdV used for veterinary vaccine-vector research because of its defined genome organization, genetic manipulability, and ability to express heterologous antigens [26,27]. The biological and engineering features of BAdV-3 that support its use as a recombinant vaccine vector are summarized in Table 2.

### 3.1. Taxonomy and Classification

BAdVs are members of the family *Adenoviridae* and comprise viruses assigned to different adenoviral taxa. Among the BAdVs investigated for recombinant vector development, BAdV-3 has received the greatest attention. Under the current International Committee on Taxonomy of Viruses (ICTV) classification, BAdV-3 is assigned to the species *Mastadenovirus bostertium*, genus *Mastadenovirus* and family *Adenoviridae*. BAdV-3 has been extensively characterized at the genomic and molecular levels and has served as the principal bovine adenoviral virus used for recombinant gene delivery and experimental vaccine-vector development [17,19,26,27].

### 3.2. Genome Organization and Virion Structure

Among bovine adenoviruses, BAdV-3 has been the most extensively characterized for recombinant vector development. Its genome organization and transcriptional map have been defined experimentally [32], and subsequent studies demonstrated successful heterologous gene insertion and development of both replication-competent and replication-defective recombinant vectors [17,18,19].

Structurally, BAdVs have a typical adenoviral capsid composed of hexon, penton base, fiber, and minor capsid or core-associated proteins. Hexon is a major antigenic determinant, while fiber contributes to cell attachment and tropism. These structural proteins are relevant to both vaccine design and vector production. For example, recombinant BAdV-3 hexon has been evaluated as a subunit vaccine candidate, and pIX has been shown to be required for successful rescue of BAdV-3 virions from full-length viral DNA [33,34]. Thus, BAdV genome organization and capsid structure directly influence vector stability, manufacturability, and antigen-delivery potential.

### 3.3. Host Range, Tropism, and Pathogenesis

BAdVs naturally infect cattle and related bovine hosts. Infection may involve respiratory or enteric tissues and can range from subclinical infection to respiratory, gastrointestinal, or systemic disease. Clinical outcome depends on viral type, host age, immune status, management conditions, and co-infections. BAdVs have been detected in association with bovine respiratory disease complex (BRDC), although their role may vary among outbreaks and herds [26,36,37].

The respiratory and enteric tropism of BAdVs is relevant to vector design because tissue targeting influences route of administration, immune induction, shedding, and safety assessment. BAdV-3 is attractive as a vector because it is bovine-derived, grows efficiently, and can be genetically modified. Nevertheless, because BAdVs naturally infect cattle, field use of BAdV-based vectors requires evaluation of pre-existing anti-BAdV immunity, vector shedding, tissue distribution, and environmental safety [26,27].

### 3.4. Immunological Features of BAdV Infection

Like other adenoviruses, BAdVs can activate innate and adaptive immune responses. Innate recognition of adenoviral infection promotes cytokine production and antigen-presenting cell activation, providing an intrinsic adjuvant effect that is useful for vaccine delivery. Recombinant BAdV-3 vectors can express antigens intracellularly, allowing antigen presentation through MHC class I and class II pathways and supporting CD8^+^ T-cell responses, CD4^+^ T-cell activation, and antibody production [3,5,20,27].

Mucosal immunity is particularly relevant because many bovine pathogens enter through respiratory or gastrointestinal surfaces. Intranasal BAdV-3 vectors expressing respiratory pathogen antigens and recombinant BAdV mucosal vaccines have shown potential to induce both mucosal and systemic immunity [21,24,27]. However, natural BAdV exposure in cattle may generate anti-vector immunity that could affect vaccine performance. Therefore, understanding BAdV immunobiology is essential for predicting the efficacy of BAdV-based vaccines in field populations.

In summary, BAdVs are biologically diverse cattle-associated adenoviruses with relevance to both disease pathogenesis and vector development. BAdV-3 is the best-characterized BAdV for recombinant vaccine applications because of its defined genome, compatibility with E1/E3-based engineering, and capacity to induce antigen-specific immune responses.

## 4. Rationale for Using BAdV as a Viral Vector

The rationale for developing BAdV-3 as a veterinary vector derives primarily from its bovine origin, genetic tractability, and ability to support recombinant antigen expression. These features provide a biological basis for further evaluation as a veterinary vaccine platform, while experimental immune outcomes are considered separately in Section 7 [15,17,18,19,20,21].

### 4.1. Non-HAdV as an Alternative Vector Platform

A major rationale for exploring BAdV is its antigenic distinction from commonly used human adenovirus (HAdV) vectors. HAdV-5 is highly characterized and immunogenic, but pre-existing anti-HAdV-5 immunity can reduce vector transduction, transgene expression, and vaccine-induced immune responses [3,9,13,38]. This limitation has stimulated the development of rare human adenovirus serotypes and non-human adenoviruses, including chimpanzee and animal adenoviruses [14,15,16].

BAdV-based influenza vaccine studies provide experimental support for this concept. BAdV vectors retained immunogenicity in the presence of high pre-existing immunity to HAdV, and BAdV-3-based H5N1 vaccine candidates demonstrated strong immunogenicity and protective activity in experimental models [22,23]. Experimental studies in mice support the potential of BAdV in this context. BAdV-based H5N1 vaccine vectors remained immunogenic in mice with high pre-existing immunity to HAdV, and subsequent BAdV-3-based H5N1 vaccination provided protection in mice [22,23]. These findings establish BAdV as an antigenically distinct alternative within the broader adenoviral vector landscape, although its performance in cattle must ultimately be considered in the context of naturally acquired anti-BAdV immunity, as discussed in Section 9.

### 4.2. Species Relevance for Veterinary Vaccine Development

BAdV-3 is particularly relevant to veterinary vaccinology because it is derived from a bovine-associated adenovirus. Its species origin distinguishes it from HAdV platforms developed primarily for human gene delivery and vaccination and provides a rational basis for evaluating BAdV-3 against cattle-associated infectious diseases [39].

The technical feasibility of this platform was demonstrated by insertion of a firefly luciferase reporter gene into the BAdV-3 E3 region, establishing that the viral genome could accommodate heterologous sequences [17]. Subsequent development of E1A-deleted replication-defective BAdV-3 provided an additional platform for controlled foreign-gene expression [19]. Importantly, recombinant BAdV-3 vectors expressing BHV-1 glycoprotein D (gD) were evaluated in cattle [20], whereas BAdV-3 vectors expressing BRSV glycoprotein G (gG), alone or together with truncated BHV-1 gD, extended the concept toward respiratory and combined-antigen vaccination [21]. Collectively, these studies establish the biological and engineering basis for BAdV-3 as a species-relevant veterinary vector.

### 4.3. Mucosal Vaccine Delivery Potential

Adenoviral vectors are also attractive for mucosal antigen delivery because they can transduce epithelial and antigen-presenting cells at respiratory or gastrointestinal surfaces [3,6]. This property is particularly relevant to veterinary pathogens that initiate infection at mucosal entry sites.

Experimental BAdV studies support this route of administration. Intranasal immunization with recombinant BAdV-3 expressing BRSV gG, alone or together with truncated BHV-1 gD, induced antigen-specific immune responses in cotton rats [21]. In addition, a recombinant bovine adenoviral mucosal vaccine expressing mycobacterial antigen 85B generated protection against tuberculosis in mice [24]. This finding provides proof of concept for mucosal BAdV vaccination in an experimental mouse model but should not be interpreted as evidence of efficacy in cattle or other livestock species.

### 4.4. Capacity for Strong Humoral and Cellular Immune Induction

Like other adenoviral vectors, BAdV vectors deliver antigen-encoding genes into host cells, allowing intracellular antigen synthesis and presentation through both MHC class I and class II pathways. This mechanism can support CD8^+^ T-cell activation, CD4^+^ T-cell responses, and antibody production [3,5,6].

Experimental studies demonstrate that this theoretical advantage can be translated into antigen-specific responses. Recombinant BAdV-3 expressing BHV-1 gD induced immune responses in cattle [20]; respiratory antigen-expressing BAdV-3 vectors induced responses following intranasal administration [21]; and BAdV-based vectors expressing influenza or mycobacterial antigens generated immunogenicity and protection in experimental animal models [22,23,24,25]. Thus, the principal rationale for BAdV-3 development is its combination of genetic tractability, bovine host relevance, and capacity for antigen delivery through systemic and mucosal vaccination strategies.

## 5. Engineering Strategies for BAdV-Based Vectors

**Figure 2:**
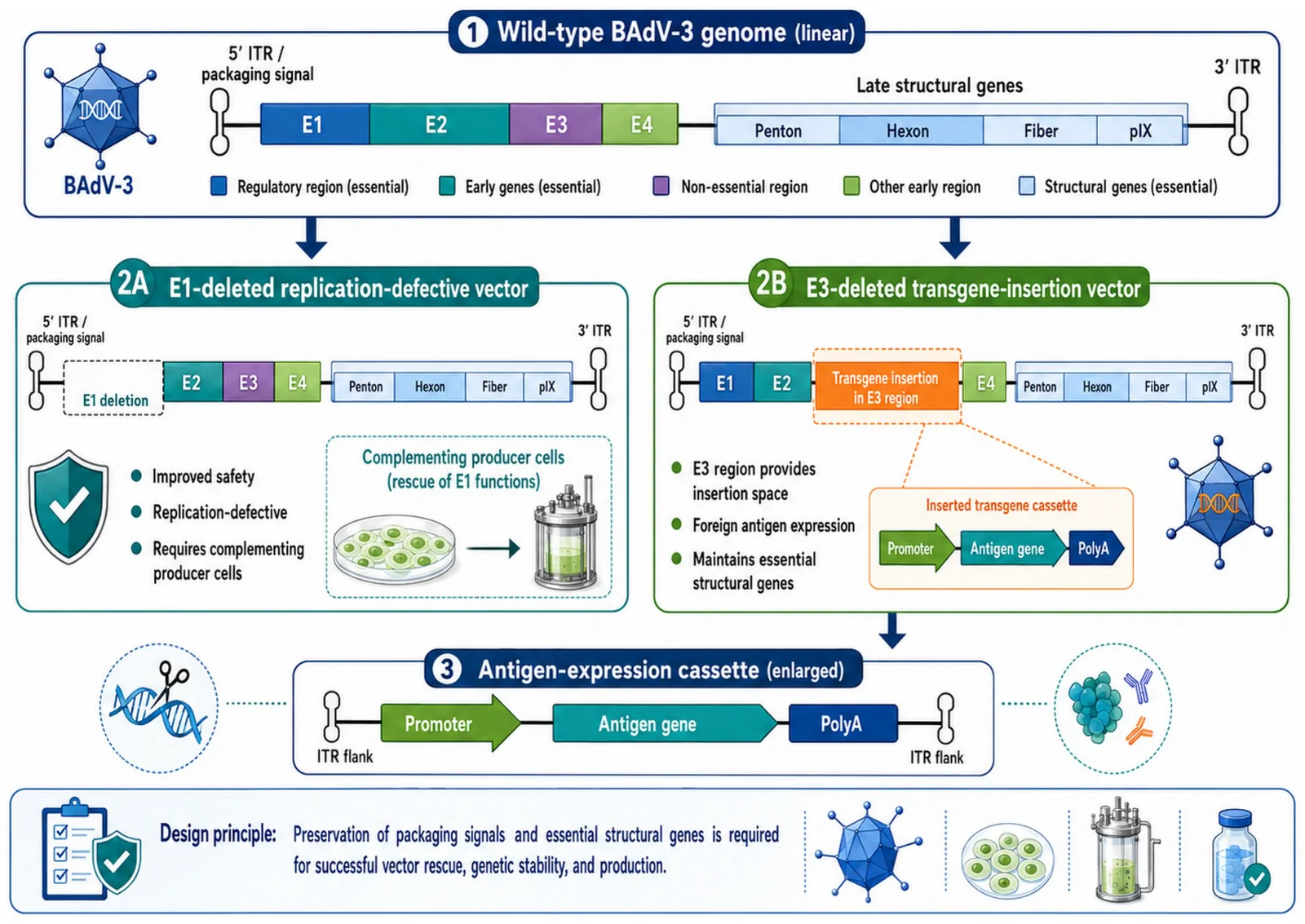
Engineering strategies for BAdV-3-based vectors. The BAdV-3 genome contains early regulatory regions (E1–E4), structural genes, and genomic regions that can be modified for recombinant vector construction. Deletion of E1, particularly E1A, restricts productive replication in normal host cells and generates replication-defective vectors that require complementing producer cells for propagation. E3 is generally dispensable for replication in cell culture and can provide a site for insertion of heterologous antigen-expression cassettes. Preservation of essential viral genes and packaging elements is required for vector rescue, genetic stability, and production. ITR, inverted terminal repeat; E1–E4, early regions 1–4; pIX, adenovirus protein IX; Poly(A), polyadenylation signal.

Engineering of BAdV-based vectors, particularly BAdV-3, relies on rational modification of the viral genome to allow foreign gene expression while preserving safety, stability, and production efficiency. Molecular studies describing the BAdV-3 genome, transcriptional map, early regions, packaging motifs, and structural proteins have provided the foundation for vector design [32,35,40,41,42,43]. Early recombinant studies confirmed that BAdV-3 could be genetically modified to express foreign genes, supporting its development as a veterinary vaccine vector [17,19,26,27]. The main genome engineering strategies used for BAdV-3 vector construction are shown in Figure 2.

### 5.1. Replication-Competent and Replication-Defective BAdV Vectors

BAdV vectors can be designed as either replication-competent or replication-defective systems. Replication-competent vectors retain essential viral genes and may provide stronger antigen expression through viral amplification, as shown by early E3-based recombinant BAdV-3 vectors expressing heterologous genes [17,18,26,44]. However, these vectors require careful biosafety evaluation because they may be associated with vector shedding, transmission, environmental dissemination, or recombination with circulating adenoviruses [20,27].

Replication-defective vectors are generated by deletion of essential replication genes, most commonly within the E1 region. E1A-deleted BAdV-3 vectors cannot complete productive replication in normal host cells and require complementing producer cell lines for propagation [19,32]. This restricted replication may provide a more favorable biosafety profile than that of replication-competent vectors. However, replication deficiency does not eliminate all vector-associated risks. Candidate vectors must still be evaluated for residual infectivity, biodistribution, vector shedding, genetic stability, and the potential generation of replication-competent recombinants during production. Thus, replication status should be considered one component of an overall biosafety assessment rather than an absolute indicator of vector safety.

### 5.2. E1-Deleted and E3-Deleted BAdV Vectors

E1 deletion is central to the generation of replication-defective BAdV vectors. Because E1 encodes regulatory proteins required for viral replication, deletion of E1A restricts productive replication in non-complementing cells and may therefore provide a more favorable biosafety profile than replication-competent vector designs. E1A-deleted BAdV-3 vectors require complementing producer cells that supply the deleted E1A functions in trans for vector rescue and propagation [19]. However, replication deficiency should not be interpreted as eliminating all vector-associated risks, because residual infectivity, biodistribution, shedding, genetic stability, and the potential generation of replication-competent recombinants must still be evaluated. This approach follows the general principle used for first-generation human adenoviral vectors [3,31].

In contrast, the E3 region is generally dispensable for adenoviral replication in cell culture and is therefore commonly used as a site for heterologous gene insertion. The first recombinant BAdV-3 expression vector incorporated a firefly luciferase reporter gene into the E3 region, demonstrating its suitability for foreign-gene insertion [17]. Subsequent E3-deleted BAdV-3 vectors were engineered to express vaccine antigens, including full-length or truncated BHV-1 glycoprotein D [18]. Thus, E1 deletion may provide a more favorable biosafety profile, whereas E3 deletion increases cloning capacity and provides a practical insertion site [44].

### 5.3. Transgene Insertion Sites and Expression Cassettes

The E3 region remains the most frequently used insertion site for BAdV-3 vectors because it can accommodate foreign genes without disrupting essential replication functions. BAdV-3 vectors have been engineered to express reporter genes and vaccine antigens from BHV-1, BRSV, influenza virus, and mycobacteria [17,18,19,20,21,23,24].

Expression cassette design is also critical. Promoter choice, antigen sequence, transcriptional termination signals, codon optimization, signal peptides, and antigen-targeting motifs can influence expression efficiency and immune response quality. Transgene stability must be assessed because large inserts, repeated sequences, or immune-modulating genes may reduce viral fitness or promote deletion during passage. Studies of BAdV-3 packaging motifs and structural proteins have identified specific viral elements involved in genome encapsidation, virion rescue, assembly, and intracellular trafficking [33,35,40,41,42,43]. Recent BAdV designs, such as universal influenza constructs using conserved nucleoprotein linked to an autophagy-inducing peptide, illustrate the potential for rational antigen engineering [25].

### 5.4. Complementing Cell Lines and Vector Production

Complementing cell lines are essential for producing replication-defective BAdV vectors. Because E1-deleted vectors cannot replicate independently, producer cells must provide the deleted E1 functions in trans. Reddy et al. established E1A-complementing cell lines that enabled rescue and propagation of E1A-deleted BAdV-3 vectors [19]. Efficient producer cells should support high-titer vector production, stable complementation, low recombination risk, and scalability for veterinary vaccine manufacturing.

Vector production also requires rigorous control of rescue, amplification, purification, and titration. Final vector preparations should be tested for infectious titer, particle concentration, antigen expression, purity, sterility, and absence of replication-competent contaminants. BAdV-3 structural proteins also influence production efficiency; for example, pIX is required for successful virion rescue, while protein V and 22K-related interactions contribute to replication, assembly, or intracellular trafficking [33,42,43].

### 5.5. Genetic Stability and Manufacturing Considerations

Genetic stability is essential for BAdV-based vaccine development. Recombinant vectors must retain their inserted antigen cassette during rescue, amplification, storage, and administration. Serial-passage studies should therefore confirm transgene retention, expression consistency, and absence of deletion variants [26,27]. Passage-related instability may occur if the insert reduces viral fitness, particularly for large or multivalent constructs.

Manufacturing scalability remains a key limitation. Replication-competent vectors may be easier to amplify but raise greater biosafety concerns, whereas replication-defective vectors may offer a more favorable biosafety profile but depend on efficient complementing cell lines. Future development should focus on improving vector yield, serum-free or scalable culture systems, purification protocols, thermostable formulations, and reliable potency assays [19,20]. Quality control should include vector identity, purity, potency, infectious titer, particle-to-infectivity ratio, genome integrity, sterility, adventitious agent testing, antigen expression, and absence of replication-competent recombinants.

In summary, BAdV vector engineering depends on selecting appropriate replication-competent or replication-defective designs, using E1 and E3 regions strategically, optimizing antigen-expression cassettes, developing reliable complementing cell lines, and ensuring genetic stability and manufacturability. Although BAdV-3 is technically feasible as a non-human adenoviral vector, further optimization of yield, stability, scale-up, and quality-control systems is required before broad veterinary application.

## 6. BAdV-Based Vectors in Veterinary Vaccine Development

BAdV-based vectors, particularly BAdV-3, have been investigated as recombinant vaccine delivery systems for veterinary and translational applications. Their development has progressed from early antigen-expression studies to respiratory vaccine candidates, influenza vaccines, mucosal immunization models, and multivalent vaccine concepts. Although less mature than human or chimpanzee adenoviral platforms, available evidence supports BAdV-3 as a promising non-human adenoviral vector for veterinary vaccine development [19,20,21,22,23,24,25,44]. Representative applications of BAdV-based vectors in veterinary and translational vaccine studies are summarized in Table 3.

### 6.1. BAdV Vectors Expressing BHV Antigens

Early BAdV vaccine studies focused on BHV-1, an important pathogen associated with infectious bovine rhinotracheitis and bovine respiratory disease. Recombinant BAdV-3 vectors expressing BHV-1 gD were evaluated directly in calves, providing one of the limited examples of target-species efficacy assessment for the platform [20]. Both replication-competent and replication-defective vectors induced gD-specific immune responses; however, partial protection following BHV-1 challenge was reported for the replication-competent BAdV-3 vector, whereas the replication-defective vector primarily demonstrated immunological priming [20]. These findings provide important proof of concept in the natural host while also indicating that further optimization is required to achieve more complete protection.

### 6.2. BAdV Vectors for Bovine Respiratory Disease-Related Pathogens

BAdV vectors are especially relevant to BRDC, which involves multiple viral and bacterial agents, including BHV-1 and BRSV. Because BRDC is multifactorial, vectors capable of inducing mucosal and systemic immunity against more than one pathogen are attractive [21]. Recombinant E3-deleted BAdV-3 vectors expressing BRSV gG alone or together with truncated BHV-1 gD induced antigen-specific immune responses following intranasal immunization in cotton rats [21]. These findings demonstrate immunogenicity and combined-antigen delivery in a non-target experimental model; however, protection against BRSV-associated disease has not yet been established by corresponding cattle challenge studies.

### 6.3. BAdV-Based Influenza Vaccine Candidates

Dose has an important influence on the immunogenicity and protective efficacy of BAdV-based vaccines. In an early H5N1 study, mice received two intramuscular immunizations with 1 × 10^8^ PFU of BAd-H5HA at a three-week interval, resulting in protective immunity even in the presence of high pre-existing HAdV-5 immunity [22]. A subsequent dose-escalation study evaluated single intranasal or intramuscular doses ranging from 1 × 10^6^ to 1 × 10^8^ PFU [23]. Complete protection against heterologous H5N1 challenge was achieved with 1 × 10^6^ PFU following intranasal vaccination and with 3 × 10^7^ PFU following intramuscular vaccination, demonstrating that both dose and route strongly influenced protective efficacy. These findings support potential dose-sparing with mucosal delivery in this mouse model; however, they should not be extrapolated directly to livestock because dose requirements may differ according to species, vector design, antigen, replication status, and route of administration.

### 6.4. BAdV Vectors for Intracellular Pathogens and Mucosal Immunity

BAdV vectors have also been evaluated for intracellular pathogens requiring strong cell-mediated immunity. A recombinant bovine adenoviral mucosal vaccine expressing mycobacterial antigen 85B generated robust protection against tuberculosis in mice, demonstrating that BAdV vectors can deliver bacterial antigens and support protective T-cell-oriented immunity [24]. However, protection against mycobacterial infection cannot be attributed solely to antigen-specific immune responses, because disease outcome is also shaped by dynamic interactions among the pathogen, the immune system, and infected host cells. Recent evidence from *Mycobacterium bovis* infection demonstrates that infection-induced changes in host–cell pathways can influence bacterial adhesion and invasion and alter cellular phenotypes. In particular, RBMX2 was identified as a host factor that facilitates *M. bovis* infection and contributes to infection-induced epithelial–mesenchymal transition through p65/MMP-9-associated signaling [45]. These findings emphasize that the evaluation of BAdV-based vaccines against mycobacterial infection should consider both vaccine-induced immunity and the host cellular environment that influences infection and disease progression. This mucosal approach is relevant because many veterinary and zoonotic pathogens enter through respiratory or gastrointestinal surfaces. Future studies should define mucosal immune correlates, compare delivery routes, and evaluate shedding and local safety in target livestock species [24].

### 6.5. Multivalent and Next-Generation Veterinary Vaccine Designs

BAdV vectors have potential for multivalent and next-generation veterinary vaccines. This is particularly relevant for BRDC, where multiple pathogens contribute to disease. The feasibility of combined antigen delivery has been shown by BAdV-3 vectors expressing BRSV gG together with truncated BHV-1 gD [21]. Future designs may include additional BRDC-associated antigens, conserved antigens, immune-enhancing sequences, or rational antigen cassettes. Universal influenza vaccine studies further illustrate how BAdV vectors can be adapted for broader antigenic coverage [25]. However, multivalent designs must be evaluated for vector capacity, transgene stability, antigen-expression balance, immune interference, vector yield, and protective efficacy in target species [21,27].

In summary, BAdV-based vectors have been applied to BHV-1 antigen delivery, BRDC-related respiratory pathogens, H5N1 and universal influenza vaccines, mucosal tuberculosis models, and multivalent vaccine concepts. Current evidence indicates that BAdV-3 is a flexible and immunogenic platform, but further optimization, target-species validation, scalable production, and rigorous safety and efficacy testing are required before broad veterinary application [20,21,22,23,24,25].

## 7. Immunological Outcomes of BAdV-Based Vaccination

**Figure 3:**
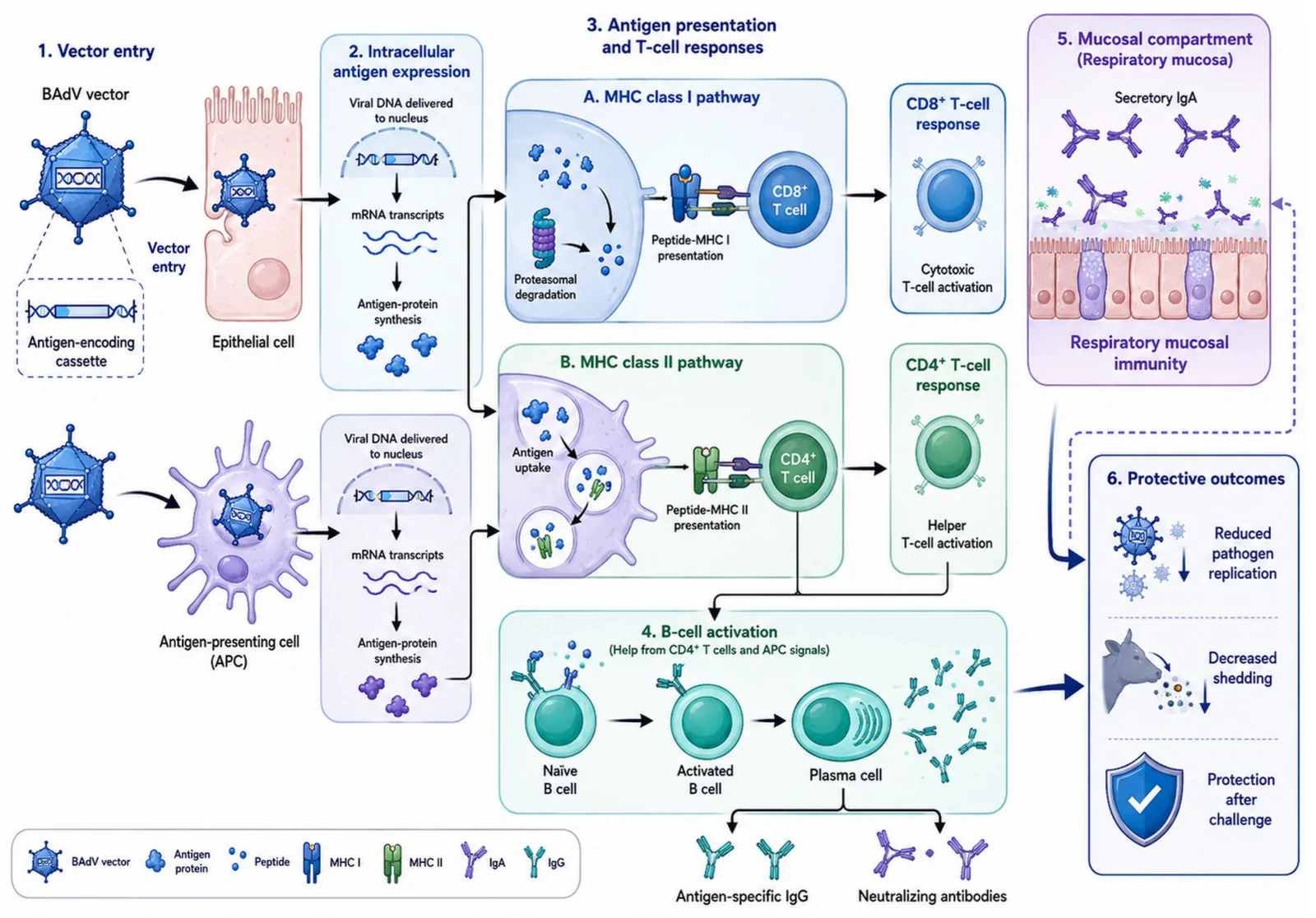
Immunological mechanisms induced by BAdV-based vaccination. Bovine adenovirus (BAdV)-based vectors deliver antigen-encoding genes into epithelial cells and antigen-presenting cells (APCs), resulting in intracellular antigen expression and antigen presentation through major histocompatibility complex (MHC) class I and class II pathways. These processes can promote CD8^+^ T-cell responses, CD4^+^ T-cell responses, B-cell activation, systemic antibody production, and mucosal immunity. Protective outcomes have been demonstrated in selected experimental models, but their extent depends on the vaccine construct, pathogen, route of administration, and experimental species.

The immunological evidence for BAdV-based vaccination is derived from studies evaluating recombinant vectors expressing viral and bacterial antigens in cattle and experimental animal models. Reported outcomes include antigen-specific antibody responses, cellular immune responses, mucosal immunity, and protection following challenge in selected models [20,21,22,23,24,25]. The broader translational limitations of the platform are discussed in Section 9. Figure 3 summarizes the principal immunological pathways associated with BAdV-mediated antigen delivery.

### 7.1. Humoral Immune Responses

Antibody responses have been among the most frequently examined outcomes of BAdV vaccination. Replication-competent and replication-defective BAdV-3 vectors expressing BHV-1 gD induced antigen-specific immune responses in cattle, providing direct evidence that the platform can function in its natural target host [20]. Recombinant BAdV-3 expressing BRSV gG, alone or together with truncated BHV-1 gD, also induced antigen-specific responses following intranasal immunization in cotton rats [21].

Influenza vaccine models provide additional evidence of humoral immunogenicity. BAdV-based H5N1 vaccine candidates generated immune responses despite high pre-existing anti-HAdV immunity [22], while a subsequent BAdV-3-based H5N1 vaccine demonstrated enhanced immunogenicity and protective efficacy at a reduced experimental dose [23]. Although systemic antibody responses have therefore been demonstrated across several models, mucosal antibody endpoints such as secretory IgA have been assessed less consistently. Standardized evaluation of nasal, bronchoalveolar, and other mucosal antibody responses would improve comparison among future studies.

### 7.2. Cellular Immune Responses

Intracellular expression of vector-encoded antigens supports MHC class I presentation and CD8^+^ T-cell activation, while uptake and processing of antigens by antigen-presenting cells can additionally promote MHC class II presentation, CD4^+^ T-cell activation, cytokine production, and immune memory [3,5,6].

Evidence for T-cell-oriented immunity has been obtained principally from experimental models. A recombinant bovine adenoviral mucosal vaccine expressing mycobacterial antigen 85B generated protective immunity against tuberculosis in mice [24]. BAdV-based universal influenza vaccine approaches using conserved influenza antigens also provided protection against influenza A and B viruses in mice and ferrets, supporting the capacity of this platform to deliver antigens intended to generate broader immune recognition [25].

Direct comparison of cellular responses across BAdV studies remains difficult because different experimental systems and immunological endpoints have been used. Future target-species studies would benefit from standardized assessment of antigen-specific CD4^+^ and CD8^+^ T cells, cytokine responses, memory phenotypes, and tissue-resident lymphocyte populations.

### 7.3. Mucosal Immune Responses

Evidence for mucosal immunogenicity is derived primarily from intranasal or other mucosal vaccination models. Recombinant BAdV-3 expressing BRSV gG and truncated BHV-1 gD induced antigen-specific responses following intranasal administration in cotton rats [21]. A separate recombinant bovine adenoviral vector expressing mycobacterial antigen 85B produced protective immunity following mucosal vaccination in mice [24].

These studies demonstrate the feasibility of delivering BAdV-vectored antigens through mucosal routes but should not be interpreted as confirmation of mucosal efficacy in cattle. Future cattle studies should characterize local antibody responses, respiratory tissue T-cell responses, mucosal cytokine profiles, and the relationship between local immunity and clinical protection.

### 7.4. Protective Efficacy in Animal Models

Protective efficacy has been demonstrated in several BAdV vaccine studies, but the experimental species differ substantially among applications and should be considered when interpreting translational relevance. For influenza, BAdV-3-based H5N1 vaccination provided protection following viral challenge in mice, including mice with experimentally induced pre-existing immunity to HAdV [22]. A subsequent dose-escalation study also demonstrated protection against heterologous H5N1 challenge in mice [23]. More recently, a BAdV-based universal influenza vaccine conferred protection against influenza A and B viruses in mice and ferrets [25]. Similarly, a recombinant bovine adenoviral mucosal vaccine expressing mycobacterial antigen 85B provided protection against *M. tuberculosis* challenge in mice [24].

Target-species efficacy data are more limited. An important exception is the evaluation of BAdV-3 vectors expressing BHV-1 gD directly in calves, in which the replication-competent vector induced partial protection following BHV-1 challenge, whereas the replication-defective vector primarily demonstrated immunological priming [20]. Thus, the current evidence base includes both target-species and non-target experimental studies, but most recent protective-efficacy data for influenza and tuberculosis derive from mouse or ferret models. Future BAdV vaccine development should therefore emphasize controlled challenge studies in the intended livestock species using clinical disease, pathogen load, lesion severity, shedding, transmission, and duration of immunity as endpoints.

## 8. Advantages of BAdV-Based Viral Vectors

BAdV-based viral vectors, particularly BAdV-3, offer several advantages for veterinary vaccine development. These advantages arise from the general properties of adenoviral vectors and the specific value of BAdV-3 as a bovine-origin, non-human adenoviral platform. Although BAdV vectors remain less mature than HAdV-5, Ad26, and chimpanzee adenoviral vectors, they have distinctive potential for veterinary vaccinology, mucosal immunization, multivalent vaccine design, and One Health preparedness [15,26,27].

### 8.1. Non-Human Adenoviral Vector Platform

A major advantage of BAdV-based vectors is their non-human adenoviral origin. Human adenoviral vectors, especially HAdV-5, are well characterized and highly immunogenic, but their effectiveness can be limited by pre-existing anti-vector immunity. Non-HAdVs have therefore been explored as alternative platforms that retain adenoviral immunogenicity while reducing limitations associated with common HAdV [3,15,16]. BAdV-3 is particularly relevant because it is antigenically distinct from HAdV and has been successfully engineered to express heterologous genes and vaccine antigens [17,19,26].

### 8.2. Reduced Impact of Pre-Existing HAdV-5 Immunity

Pre-existing immunity to HAdV-5 can reduce vector entry, antigen expression, and vaccine-induced immune responses, which has driven interest in rare human and non-human adenoviral vectors [3,9,13]. BAdV-based vectors may avoid this limitation because anti-HAdV immunity is less likely to neutralize BAdV. This advantage has been demonstrated in BAdV-based influenza vaccine studies, where immune responses were maintained despite high pre-existing immunity to HAdV [14,22,23]. However, natural BAdV exposure in cattle may generate anti-BAdV immunity; therefore, seroprevalence and vector-neutralizing antibodies should be evaluated in target herds.

### 8.3. Suitability for Veterinary Vaccine Development

BAdV vectors are well suited for veterinary vaccine development because they are derived from a bovine-associated virus and can be engineered to express antigens from cattle pathogens. This species relevance distinguishes BAdV from human adenoviral platforms. Early BAdV-3 vectors expressing BHV-1 gD induced immune responses in cattle, demonstrating feasibility in the natural host species [20]. Subsequent BAdV-3 vectors expressing BRSV and BHV antigens further supported their potential for veterinary respiratory vaccine development [21].

### 8.4. Strong Immune Response Induction

BAdV vectors can induce both humoral and cellular immunity. Like other adenoviral vectors, they deliver antigen-encoding genes into host cells, enabling intracellular antigen expression, MHC class I presentation, CD8^+^ T-cell responses, MHC class II presentation, CD4^+^ T-cell activation, and antibody production [3,5,6]. This immunological profile is useful for veterinary vaccines because protection against many pathogens requires both antibody-mediated and cell-mediated responses. Studies using BAdV vectors against BHV, BRSV, influenza, and mycobacterial antigen 85B illustrate the immunological flexibility of this platform [20,21,23,24,25].

### 8.5. Non-Integrating Genome and Transient Antigen Expression

Another safety-related advantage is that adenoviral genomes generally remain episomal rather than integrating into the host genome. This allows transient antigen expression, which is usually sufficient for vaccination, while reducing concerns associated with insertional mutagenesis [3,6]. This feature is especially relevant for food-producing animals, where safety and regulatory acceptability are important. Nevertheless, non-integration does not remove the need to evaluate vector shedding, recombination, genetic stability, and replication competence.

### 8.6. Future Potential for Mucosal Vaccination

BAdV-based vectors have been investigated as potential platforms for mucosal antigen delivery, particularly against respiratory pathogens. Intranasal BAdV-3 vectors expressing BRSV gG and truncated BHV-1 gD induced antigen-specific immune responses in cotton rats [21], while a recombinant BAdV mucosal vaccine expressing mycobacterial antigen 85B provided protection against tuberculosis in mice [24]. These studies demonstrate the feasibility of mucosal BAdV vaccination in experimental models but do not establish efficacy in cattle. Further target-species studies are required to define appropriate dose and route, local and systemic immune correlates, duration of immunity, shedding, local reactogenicity, and protective efficacy before mucosal BAdV vaccination can be considered for veterinary application.

### 8.7. Future Potential for Multivalent Vaccine Development

Recombinant BAdV-3 vectors co-expressing BRSV gG and truncated BHV-1 gD demonstrate the technical feasibility of delivering more than one antigen from a single vector [21]. This observation provides a rationale for future development of multivalent BAdV vaccines, particularly for multifactorial diseases such as bovine respiratory disease complex. However, multivalent efficacy has not yet been demonstrated in cattle, and candidate constructs will require evaluation of insert capacity, genetic stability, relative antigen expression, immune interference, vector yield, dose requirements, and protective efficacy in the target species.

### 8.8. Potential One Health-Oriented Applications

BAdV vectors could potentially contribute to future One Health-oriented vaccine strategies because they have been experimentally adapted to antigens from pathogens with relevance beyond conventional bovine diseases, including influenza and tuberculosis [23,24,25]. However, the use of a BAdV vector against a zoonotic pathogen does not itself demonstrate a One Health benefit. Such applications would require evidence that vaccination contributes to measurable outcomes such as reduced pathogen circulation, transmission, disease burden, antimicrobial use, or spillover risk. Accordingly, One Health applications should currently be considered a future research direction rather than an established advantage of the BAdV platform.

### 8.9. Overall Synthesis

Overall, current evidence supports BAdV-3 as an experimentally tractable, bovine-origin adenoviral vector capable of heterologous antigen expression and induction of antigen-specific immune responses. Mucosal vaccination, multivalent antigen delivery, and One Health-oriented applications represent potentially valuable future directions rather than established veterinary uses. Their practical relevance will depend on confirmation of target-species efficacy and duration of immunity, characterization of pre-existing anti-BAdV immunity, evaluation of vector shedding and recombination, development of scalable and reproducible manufacturing processes, and fulfillment of veterinary regulatory requirements [15].

## 9. Current Challenges and Limitations

Despite successful recombinant engineering and proof-of-concept vaccination studies, BAdV-based vectors remain an early-stage vaccine platform. The principal barriers to translation concern the depth of the evidence base, validation in target livestock species, vector-specific immunity, production technology, biosafety, and progression toward standardized veterinary vaccine manufacture. Table 4 summarizes these major limitations.

### 9.1. Limited Research Compared with HAdV, Ad26, and ChAd Vectors

The BAdV literature is substantially smaller than that available for established HAdV, Ad26, and chimpanzee adenoviral platforms. Moreover, recombinant BAdV research has been concentrated predominantly on BAdV-3 [10,11,12,17,19]. Relatively few studies have systematically compared vector dose, administration route, duration of immunity, immune correlates, genetic stability, and production performance. Consequently, the present evidence supports BAdV-3 as a proof-of-concept veterinary vector rather than as a mature vaccine platform.

### 9.2. Limited Data in Target Animal Species

Target-species evidence remains one of the most important gaps. BAdV-3 vectors expressing BHV-1 gD have been evaluated in cattle and demonstrated antigen-specific immune responses, providing direct proof of concept in the natural host [20]. In contrast, several subsequent respiratory, influenza, and tuberculosis vaccine studies used cotton rats, mice, ferrets, or other experimental models [21,22,23,24,25].

These models provide valuable information on immunogenicity and biological mechanisms but cannot reproduce all aspects of cattle immunity, respiratory physiology, vector tropism, naturally acquired immunity, husbandry conditions, or pathogen exposure. Target-species trials should therefore determine dose–response relationships, clinical efficacy, duration of protection, and field performance under representative production conditions [26,27].

### 9.3. Need for Efficient Producer Cell Lines

Replication-defective BAdV vectors depend on producer cells capable of providing deleted viral functions in trans. E1A-complementing cells have enabled rescue and propagation of E1A-deleted BAdV-3 vectors [19], but producer-cell systems remain considerably less developed than those available for established human adenoviral vectors.

For veterinary application, producer cells must support reproducible high-titer production while maintaining vector identity and genetic stability. Their suitability for serum-free culture, suspension adaptation, large-scale bioreactors, and economical vaccine manufacture will be important determinants of whether replication-defective BAdV vectors can progress beyond laboratory-scale production [19].

### 9.4. Vector Shedding, Recombination Risk, and Biosafety

Replication status has direct implications for BAdV biosafety. Replication-competent vectors may permit greater amplification in the vaccinated host but require evaluation of shedding, transmission, persistence, and environmental dissemination. Replication-defective vectors reduce productive replication in normal host cells but do not eliminate the need to assess residual infectivity, biodistribution, shedding, genetic stability, or the generation of replication-competent recombinants during production [19,20].

These issues should be investigated using vector-specific assays capable of distinguishing administered recombinant vectors from circulating adenoviruses. Detailed regulatory and environmental considerations associated with these risks are discussed separately in Section 10.

### 9.5. Pre-Existing and Maternally Derived Immunity to BAdV-3 in Cattle

The ability of BAdV-based vectors to circumvent pre-existing immunity against common human adenoviruses represents an important advantage of this platform [22,23]. However, this benefit may be less pronounced in cattle, where natural exposure to bovine adenoviruses can generate vector-specific neutralizing antibodies and cellular immune responses before vaccination. In young calves, an additional source of pre-existing immunity is the passive transfer of anti-BAdV-3 antibodies through colostrum. Longitudinal serological monitoring of dairy calves demonstrated maternally derived BAdV-3-neutralizing antibodies, with antibody levels beginning to decline from the second month of life and reaching their lowest level at approximately six months of age [46]. Earlier seroepidemiological observations similarly showed that maternal antibodies against BAdV-1 to BAdV-3 were common in calves and had largely disappeared by approximately five months of age [47]. Such pre-existing or maternally derived antibodies could theoretically neutralize vaccine particles before efficient cellular entry, thereby reducing vector transduction, transgene expression, immune priming, and the effectiveness of homologous booster vaccination. Nevertheless, the prevalence, magnitude, and functional consequences of anti-BAdV immunity remain insufficiently characterized across cattle populations, and the extent to which maternally derived antibodies interfere specifically with recombinant BAdV-3 vaccination has not been systematically determined.

Importantly, pre-existing BAdV-3-specific immunity does not appear to completely prevent vaccine responsiveness. Intranasal administration of replication-competent BAdV-3 vectors expressing full-length or truncated BHV-1 gD induced antigen-specific antibodies in serum and nasal secretions, lymphoproliferative responses, and partial protection against BHV-1 challenge in calves that already possessed BAdV-3-specific antibodies [48]. These findings suggest that mucosal delivery and/or replication-competent vector design may preserve immunogenicity in the presence of pre-existing vector immunity, although the specific contribution of maternally derived antibodies was not evaluated independently. Future studies should therefore characterize anti-BAdV immunity across different ages, production systems, geographical regions, and BAdV types, and directly compare calves with defined high and low maternally derived antibody titers. Such studies are needed to determine the effects of vector-specific immunity on transduction efficiency, antigen expression, systemic and mucosal immune responses, booster efficacy, and protective immunity. If clinically relevant interference is demonstrated, potential strategies may include optimized vaccination timing, heterologous prime–boost regimens, alternative BAdV types, capsid modification, or sequential use of different viral-vector platforms.

### 9.6. Limited Manufacturing Scale-Up Data

Manufacturing experience with BAdV vectors remains limited compared with HAdV-5, Ad26, and ChAd platforms, for which production, purification, formulation, and quality-control systems are substantially more established [10,11,12]. Translation of BAdV vaccines will require scalable upstream and downstream processes capable of generating consistent infectious titers and particle quality at costs appropriate for livestock vaccination.

Priority areas include serum-free suspension culture, high-yield producer cells, scalable purification, formulation stability, and validated quality-control assays for vector identity, infectious titer, particle concentration, transgene integrity, antigen expression, purity, sterility, potency, and absence of unwanted replication-competent contaminants.

### 9.7. Lack of Widely Used Commercial BAdV-Vector Vaccines

No BAdV-vector vaccine has yet achieved widespread routine veterinary use. The current literature consists primarily of vector-development studies, proof-of-concept experiments, and a limited number of target-species evaluations [20,21,22,23,24,25]. Translation will therefore require coordinated evidence linking reproducible manufacturing with target-species efficacy and safety.

Accordingly, BAdV should presently be regarded as an emerging veterinary vector platform. Its scientific potential is supported by successful recombinant engineering and experimental vaccination studies, but its commercial value cannot be established until candidate vaccines are evaluated through development pathways comparable to those used for other veterinary biological products.

### 9.8. Overall Interpretation

BAdV-3 can be engineered to express heterologous antigens and has demonstrated immunogenicity across several animal models. Protective efficacy has been reported in mice and ferrets for influenza and tuberculosis vaccine candidates, while limited target-species challenge studies have demonstrated partial protection against BHV-1 in calves [20,22,23,24,25].

## 10. Biosafety and Regulatory Considerations

Biosafety and regulatory assessment are essential for developing BAdV-based vectors as veterinary vaccines, especially because these products may be administered to large livestock populations under field conditions. Key concerns include replication competence, host restriction, shedding, transmission, recombination, genetic stability, target-species safety, and compliance with veterinary biological product regulations. These issues are particularly important for food-producing animals, where animal health, environmental safety, public confidence, and trade implications must be considered.

### 10.1. Replication Competence and Host Restriction

Replication competence is a major biosafety determinant. Replication-competent BAdV vectors may enhance antigen expression and immune stimulation, especially after mucosal delivery, but they require stricter evaluation because of possible replication, shedding, transmission, and persistence in vaccinated populations [20]. In contrast, replication-defective vectors, such as E1A-deleted BAdV-3, cannot complete productive replication in normal host cells and require complementing producer cells, making them generally more favorable from a biosafety perspective [19]. Host restriction must also be assessed because BAdV vectors should show predictable tropism, limited replication outside intended conditions, and no uncontrolled spread to non-target animals or humans [15].

### 10.2. Environmental Shedding and Transmission

Environmental shedding is a key concern for live recombinant veterinary vaccines. BAdV vectors may be shed through nasal secretions, feces, saliva, urine, milk, or other materials depending on vector design, tropism, and route of administration. This is especially relevant for intranasal or oral vaccines, where vector detection in respiratory or gastrointestinal secretions may occur [21,24]. Shedding should be evaluated for magnitude, duration, infectivity, environmental persistence, and transmission to contact animals. Future studies should monitor nasal swabs, feces, blood, relevant tissues, and pen-mates under field-relevant conditions.

### 10.3. Recombination and Genetic Stability

Recombination and genetic instability are important biosafety and quality-control issues. Replication-defective BAdV vectors may theoretically regain replication competence through recombination with complementing sequences in producer cells or with naturally circulating BAdVs in cattle. Therefore, producer cell lines should minimize sequence overlap with the vector genome, and vaccine lots should be tested for replication-competent BAdV contaminants [19]. Genetic stability should be confirmed through serial passage, sequencing, transgene-retention assays, antigen-expression testing, and detection of recombinants. Structural requirements must also be preserved; for example, BAdV-3 pIX is important for successful virion rescue [33].

### 10.4. Safety Assessment in Target Animal Species

Safety testing in target animal species is essential because small-animal models cannot fully predict cattle responses. Target-species studies should assess local and systemic reactions, clinical signs, body temperature, feed intake, hematology, biochemistry, tissue distribution, shedding, and pathology [20]. Evaluation should include relevant age groups and physiological conditions, especially calves, pregnant or lactating animals, and stressed or immunologically compromised animals when appropriate. Dose-escalation and route-comparison studies are also needed, particularly for intranasal vaccines where local respiratory safety, inflammation, lung lesions, and exacerbation of concurrent respiratory disease must be assessed.

### 10.5. Regulatory Requirements for Veterinary Viral Vector Vaccines

Veterinary viral vector vaccines must meet requirements for quality, safety, efficacy, genetic stability, and environmental risk assessment. For BAdV-based vaccines, regulatory development should include well-characterized master and working seed stocks, defined producer cell lines, validated manufacturing processes, and standardized release assays. Key release tests should include identity, purity, potency, infectious titer, particle concentration, particle-to-infectivity ratio, transgene identity, antigen-expression potency, sterility, adventitious agent testing, and absence of replication-competent contaminants for replication-defective vectors [26,27].

Efficacy should be supported by immunogenicity and, where possible, target-species challenge studies. For bovine respiratory vaccines, relevant endpoints include reductions in clinical disease, respiratory signs, pathogen load, lung lesions, shedding, transmission, and mortality. Because bovine respiratory disease is multifactorial, field trials may ultimately be required to confirm practical protection under commercial conditions.

Overall, BAdV-based vectors require a balanced biosafety and regulatory strategy integrating vector design, production control, shedding and transmission assessment, recombination and genetic stability testing, target-species safety, environmental evaluation, and efficacy studies. Replication-defective vectors may offer safety advantages, whereas replication-competent vectors may provide stronger immunogenicity but require more extensive transmission and environmental-risk assessment.

## 11. Comparative Perspective with Other Viral Vector Platforms Used in Veterinary Vaccination

**Figure 4:**
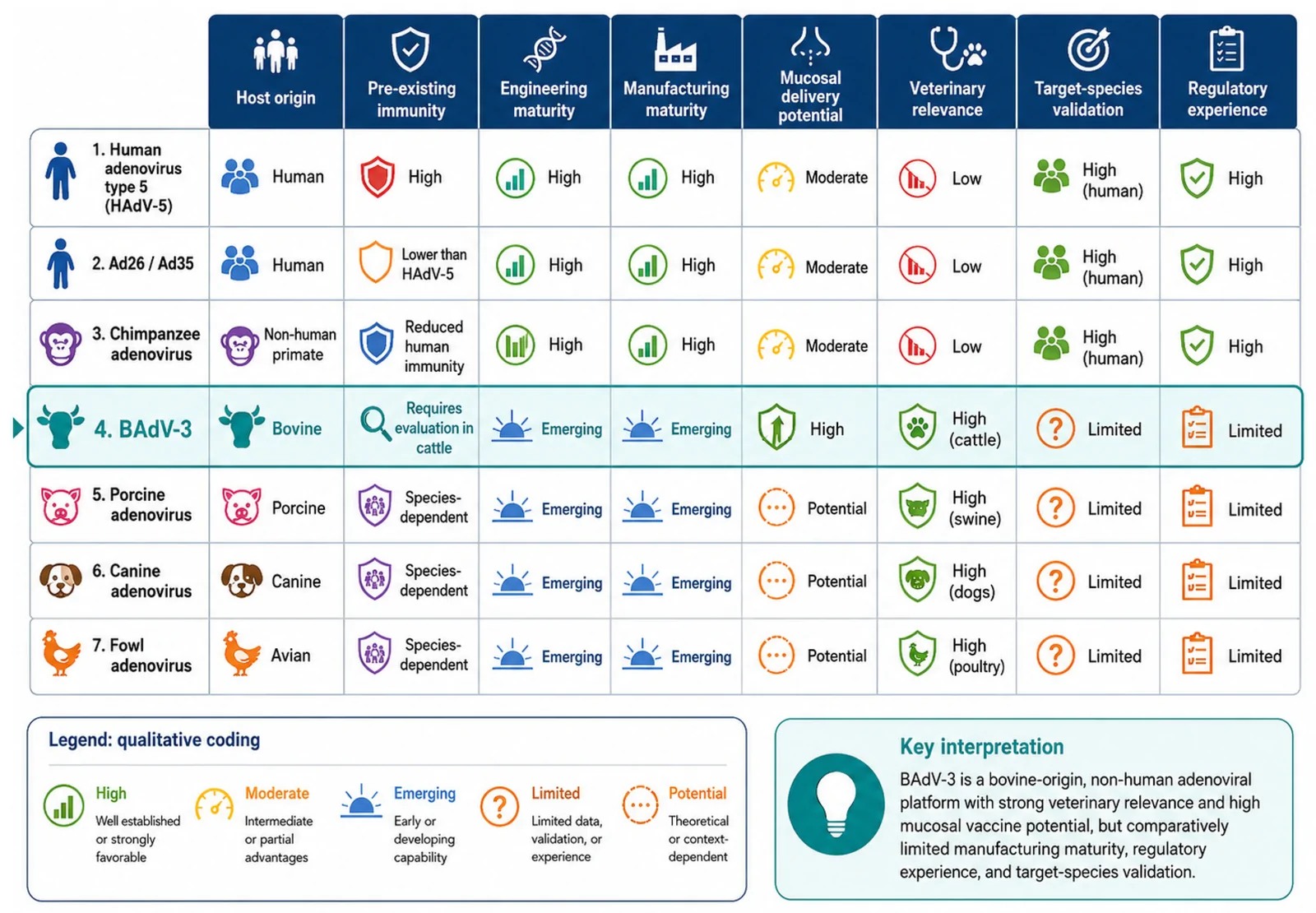
Comparative positioning of BAdV among adenoviral vector platforms. BAdV-3 is compared with HAdV-5, human adenovirus types 26 and 35 (Ad26/Ad35), chimpanzee adenoviruses, and selected animal adenoviruses according to host origin, pre-existing vector immunity, engineering maturity, manufacturing maturity, mucosal-delivery potential, veterinary relevance, target-species validation, and regulatory experience. The comparative categories are qualitative and reflect the relative maturity and available evidence for each platform rather than direct head-to-head experimental comparisons.

Comparison with established human and animal adenoviral vectors helps define the potential niche of BAdV-3 more precisely. Adenoviral platforms share general features such as efficient transgene delivery and strong immunogenicity but differ substantially in host origin, baseline vector immunity, veterinary relevance, production maturity, and translational experience [3,14,15,16,49]. Figure 4 and Table 5 summarize the comparative positioning of BAdV-3 within this broader vector landscape.

### 11.1. Comparison with Human Adenoviral Vector Platforms

HAdV-5 represents a highly characterized adenoviral vector with extensive experience in gene transfer and vaccine development [3,5,9,49]. Its major technical strengths include well-established vector engineering and production systems, whereas widespread pre-existing HAdV-5 immunity can reduce vector transduction and vaccine responses in human populations [13,50,51].

BAdV-3 differs principally in host origin and intended application. BAdV-based influenza studies demonstrated immunogenicity despite high anti-HAdV immunity [22,23], supporting antigenic distinction from HAdV-5. Conversely, HAdV-5 has a much more mature manufacturing and translational infrastructure. Thus, the comparative value of BAdV is not that it universally outperforms HAdV-5, but that it provides a distinct non-human vector that can be developed specifically for veterinary applications.

To better define the position of BAdV-3 among veterinary viral-vector platforms, a comparative assessment should consider not only host origin and immunogenicity but also biological and technological characteristics that directly influence vaccine development and field application. These include genome size and organization, experimentally demonstrated insert capacity, replication status, pre-existing or maternally derived vector immunity, production efficiency, and suitability for mucosal delivery. Such parameters can affect antigen-design flexibility, vaccine dose, safety, manufacturing scalability, cost, and performance in target animal populations. Table 6 therefore compares BAdV-3 with representative veterinary viral-vector platforms, including herpesvirus of turkey (HVT), fowlpox virus, Newcastle disease virus (NDV), Capripoxvirus, and pseudorabies virus (PRV). Because insert capacity, production yield, and vector-specific immunity may vary according to vector construct, producer-cell system, host population, geographical region, and experimental conditions, the values summarized in the table should be interpreted as representative characteristics rather than fixed platform-specific limits.

### 11.2. Comparison with Ad26 and Ad35

Ad26 and Ad35 were developed partly to address the high baseline immunity associated with HAdV-5. Both have been evaluated as alternative human adenoviral platforms, with Ad26 progressing substantially further through clinical development and licensed human vaccine applications [10,11,12,50,51,77,78,79].

Relative to these vectors, BAdV-3 has considerably less translational and manufacturing experience but greater conceptual relevance to cattle vaccination. Consequently, Ad26 and Ad35 provide useful benchmarks for vector engineering, immunogenicity, and production, whereas BAdV-3 represents a host-associated platform whose potential value lies primarily in veterinary rather than human vaccination [26,27].

### 11.3. Comparison with ChAd Vectors

Chimpanzee adenoviruses (ChAd) represent another non-human strategy for reducing interference from immunity to common HAdV. Their development, including deployment during the SARS-CoV-2 pandemic, demonstrates that non-human adenoviral vectors can be engineered, manufactured at scale, and advanced through clinical and regulatory pathways [10,11,80,81,82].

BAdV and ChAd therefore share the general characteristic of non-human adenoviral origin but differ markedly in development context. ChAd platforms have been optimized primarily for human vaccination, whereas BAdV-3 is being investigated principally for cattle-associated and veterinary applications [15]. ChAd consequently provides an important technological comparator rather than a directly competing veterinary platform.

### 11.4. Comparison with Porcine, Canine, and Fowl Adenovirus Vectors

Porcine, canine, and fowl adenoviruses have also been investigated as host-associated vector platforms for swine, companion animals, and poultry, respectively [15,16]. Together with BAdV, these systems illustrate the broader concept of adapting animal adenoviruses for species-relevant vaccination.

Their principal distinctions arise from host origin and intended target species. BAdV is most directly relevant to cattle; porcine adenoviruses (PAdV) to swine; canine adenoviruses (CAdV) to dogs; and fowl adenoviruses (FAdV) to poultry. Compared with established human adenoviral vectors, however, these veterinary platforms generally have smaller evidence bases and less standardized production systems. Direct comparative studies among animal adenoviral vectors remain limited.

### 11.5. Comparative Synthesis Across Key Criteria

BAdV-3 occupies a distinct position within the adenoviral vector spectrum. HAdV-5, Ad26, and ChAd have the advantages of extensive engineering knowledge, established production technologies, and substantially greater translational experience. BAdV-3, in contrast, offers bovine host relevance and direct compatibility with veterinary vaccine development [11,15,16,17,18,19,27].

Route of administration may represent an additional point of differentiation. Intranasal BAdV-3 vaccination has been investigated in respiratory antigen-delivery models [21], and bovine adenoviral vectors have also been evaluated for mucosal tuberculosis vaccination in mice [24]. These data justify further evaluation of BAdV for livestock respiratory vaccination, but they do not yet establish superior mucosal performance relative to other adenoviral platforms.

The most informative future comparisons should therefore use common experimental endpoints rather than relying on theoretical platform advantages. Relevant criteria include vector dose, transgene expression, systemic and mucosal immune responses, challenge protection, duration of immunity, shedding, manufacturing yield, and performance in the presence of vector-specific immunity.

Taken together, no single viral-vector platform is optimal for all veterinary applications. HVT and fowlpox vectors benefit from extensive poultry vaccine experience; NDV provides efficient respiratory and mass-delivery options; Capripoxvirus offers strong ruminant host relevance and multivalent capacity; and PRV demonstrates the feasibility of host-adapted multivalent vectors in swine. In this landscape, BAdV-3 is distinguished by its bovine origin, defined genome organization, E1/E3-based genetic tractability, availability of replication-competent and replication-defective designs, and potential for respiratory mucosal antigen delivery. Its principal disadvantages are the comparatively limited cattle efficacy data, incomplete knowledge of naturally acquired anti-BAdV immunity, unresolved shedding and recombination questions, and less mature manufacturing and regulatory experience. BAdV-3 should therefore be regarded as a complementary emerging veterinary vector whose most appropriate niche may ultimately lie in cattle-specific respiratory, mucosal, or multivalent vaccination rather than as a universal alternative to established veterinary viral-vector platforms.

### 11.6. Overall Interpretation

BAdV-3 should be positioned as a complementary veterinary adenoviral platform rather than a replacement for established human or chimpanzee adenovirus vectors. Its principal differentiating feature is its bovine origin and consequent relevance to cattle vaccine development, whereas its ultimate competitive value will depend on direct comparative evidence in target animal species.

## 12. Future Perspectives

**Figure 5:**
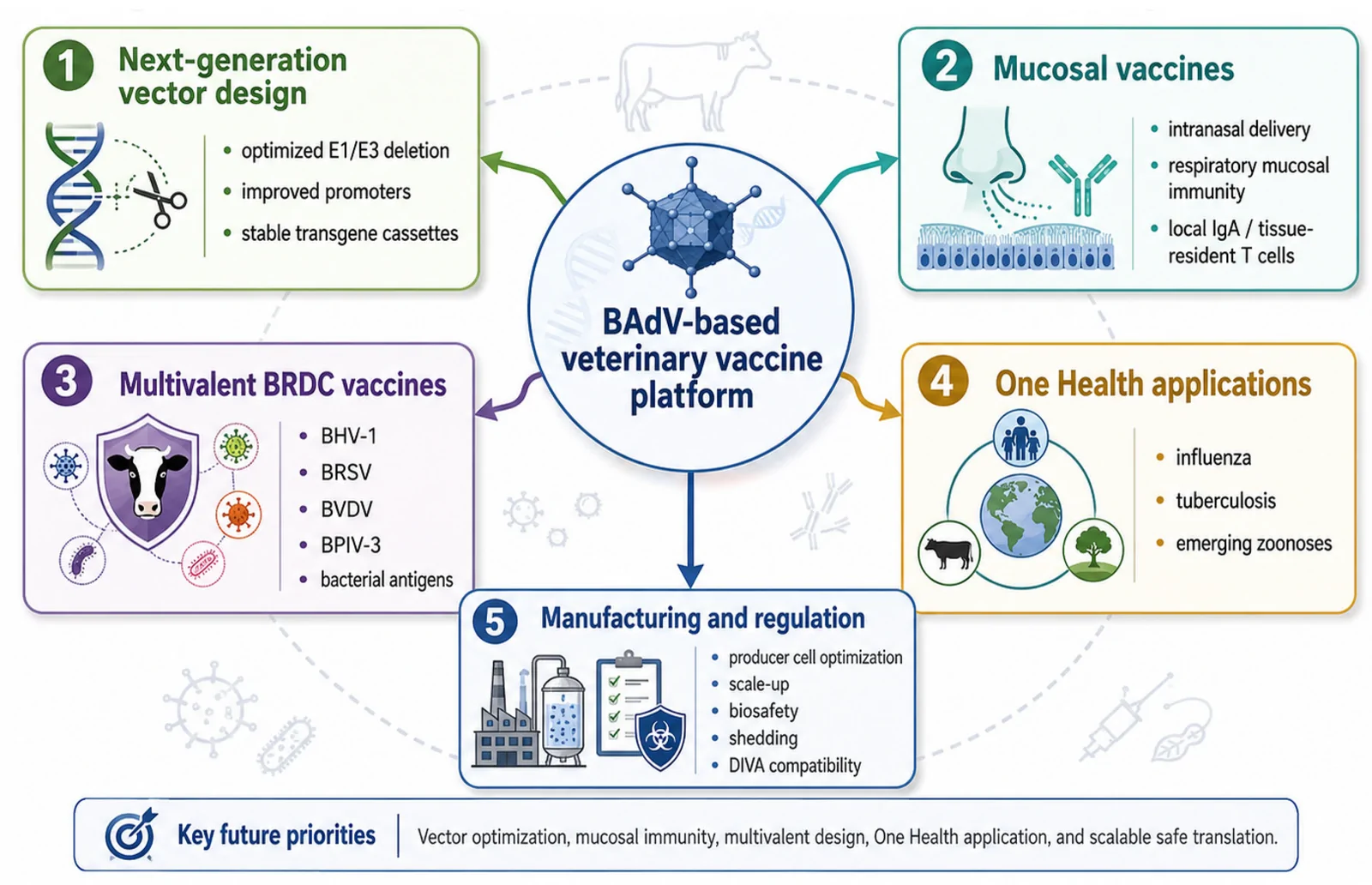
Future development framework for BAdV-based veterinary vaccines. Future development of BAdV-based vectors should prioritize next-generation vector engineering, optimization of mucosal vaccination, target-species evaluation, multivalent vaccine design for BRDC, One Health-oriented applications, scalable manufacturing, biosafety assessment, and regulatory development. Potential BRDC targets include BHV-1, BRSV, BVDV, and bovine parainfluenza virus type 3 (BPIV-3). Integration with differentiation of infected from vaccinated animals (DIVA) strategies may facilitate surveillance-based disease-control programs. E1, early region 1; E3, early region 3; IgA, immunoglobulin A.

Future BAdV research should move beyond additional demonstrations of vector feasibility and focus on experiments that directly address veterinary translation. The highest priorities are refinement of vector design, dose and route optimization, target-species challenge studies, multivalent antigen delivery, scalable production, and comparative evaluation against alternative vaccine platforms. Figure 5 summarizes these development priorities.

### 12.1. Development of Next-Generation BAdV Vectors

Future BAdV-3 engineering should emphasize predictable antigen expression, genetic stability, and reproducible production. Early studies established the feasibility of foreign-gene insertion into the E3 region and production of E1A-deleted replication-defective vectors [17,18,19]. These systems provide a foundation for optimization of promoters, insertion sites, codon usage, antigen-trafficking signals, and expression-cassette architecture.

Next-generation constructs may incorporate conserved antigens, multi-epitope designs, or immune-modulating elements when supported by a clear biological rationale. The use of conserved influenza antigens in recent BAdV vaccine studies illustrates how vector engineering can be extended beyond single-strain antigen delivery toward broader immune coverage [25].

### 12.2. Further Evaluation of Mucosal BAdV Vaccination

Future mucosal studies should shift from demonstrating feasibility toward defining dose, route, immune correlates, and protective efficacy in the intended host. Intranasal BAdV-3 vectors expressing BRSV gG and truncated BHV-1 gD provide a proof-of-concept respiratory delivery model [21], while mucosal delivery of a BAdV vector expressing mycobacterial antigen 85B produced protection in mice [24].

In cattle, studies should compare intranasal and parenteral administration using standardized measurements of nasal and bronchoalveolar antibodies, mucosal IgA, tissue-resident T cells, local cytokine responses, and clinical outcomes after pathogen challenge. Such experiments would determine whether mucosal delivery provides a measurable advantage over conventional vaccination rather than assuming superiority from route of administration alone.

### 12.3. Development and Validation of Multivalent BAdV Vaccines

BRDC represents a logical target for multivalent BAdV development because multiple viral and bacterial agents contribute to disease. BAdV-3 vectors expressing BRSV gG alone or together with truncated BHV-1 gD demonstrate that combined antigen expression is technically feasible [21].

Future constructs could incorporate antigens from additional BRDC-associated pathogens, provided that insert size, expression balance, vector stability, and immune interference are experimentally evaluated [26,37]. Multivalent candidates should progress through sequential studies of antigen expression, immunogenicity, dose optimization, and challenge protection before field evaluation.

### 12.4. BAdV Vectors for Emerging Zoonotic Diseases

BAdV may also be explored as a rapidly adaptable antigen-delivery platform for pathogens at the animal–human interface. H5N1 and universal influenza studies demonstrate that bovine adenoviral vectors can be adapted to antigens of zoonotic and pandemic relevance [22,23,25].

For preparedness applications, the major requirement will be development of standardized vector backbones and cloning procedures that allow new antigen cassettes to be introduced without repeatedly rebuilding the production platform. Such an approach could support more rapid preclinical assessment of candidate vaccines against newly emerging pathogens.

### 12.5. Integration with DIVA Vaccine Strategies

Recombinant BAdV vaccines may also be compatible with DIVA approaches because defined protective antigens can be expressed selectively while other pathogen antigens remain available as diagnostic markers. Candidate vaccines expressing selected glycoproteins, conserved proteins, or subunits could therefore be paired with diagnostic assays directed against non-vaccine antigens.

This approach could be particularly useful for diseases in which vaccination must be integrated with surveillance, movement control, or eradication programs. Future studies should validate both the vaccine component and companion diagnostic assay under experimental and field conditions before a DIVA claim can be established.

### 12.6. Evaluation of One Health-Oriented Applications

One Health applications should be defined by measurable effects on animal and public health rather than by the vector platform alone. Influenza and tuberculosis studies demonstrate that BAdV vectors can deliver antigens from pathogens with relevance beyond conventional bovine disease [24,25].

Future studies could therefore prioritize livestock pathogens whose control may reduce zoonotic transmission, antimicrobial use, production losses, or pathogen circulation at the human–animal interface. Linking vaccine efficacy studies with epidemiological and transmission endpoints would provide stronger evidence for a One Health contribution than immunogenicity alone.

### 12.7. Producer Cell Lines and Scalable Manufacturing

Manufacturing research should proceed in parallel with biological optimization rather than after selection of a final vaccine candidate. E1A-complementing cells have established the feasibility of producing replication-defective BAdV-3 [19], but future work should optimize cell growth, vector yield, suspension adaptation, serum-free media, purification recovery, formulation stability, and batch consistency.

Development-stage quality-control panels should include infectious titer, particle concentration, vector identity, transgene integrity, antigen expression, genetic stability, sterility, purity, potency, and testing for replication-competent recombinants [27]. Establishing these parameters early would facilitate comparison among candidate constructs and support subsequent manufacturing scale-up.

### 12.8. Comparative Studies with Other Adenoviral Platforms

Direct comparison with alternative adenoviral and non-adenoviral vaccine platforms will be important for identifying where BAdV provides a genuine practical advantage. Comparative studies should use matched antigens and experimental conditions whenever possible and evaluate dose requirement, systemic and mucosal immunity, challenge efficacy, safety, shedding, manufacturing yield, and performance in animals with pre-existing vector immunity.

For veterinary translation, these comparisons should ultimately be performed in the intended livestock species. Such studies would clarify whether BAdV-3 is best suited as a primary vaccine vector, mucosal booster, component of heterologous prime–boost regimens, or specialized platform for multivalent respiratory vaccination.

### 12.9. Overall Future Outlook

The next phase of BAdV research should therefore prioritize decision-enabling studies rather than additional proof-of-concept demonstrations. Progress toward veterinary application will depend on integrating optimized vector engineering with quantitative target-species efficacy, dose selection, reproducible manufacturing, and comparative performance data. This evidence will determine which proposed BAdV applications warrant progression toward field evaluation and eventual veterinary product development.

## 13. Conclusions

BAdV-based vectors, particularly BAdV-3, constitute an emerging non-human adenoviral platform with demonstrated capacity for recombinant antigen expression and experimental vaccination. BAdV-3 has been engineered to express antigens from BHV-1, BRSV, influenza virus, and Mycobacterium tuberculosis, with immune responses demonstrated in cattle and protective efficacy reported principally in experimental influenza and tuberculosis models. These studies establish the biological feasibility of the platform but do not yet demonstrate broad efficacy in target livestock populations. The immediate priority is therefore to translate proof-of-concept findings into well-controlled cattle studies addressing dose, route, protection, duration of immunity, and vector-specific immunity while developing reproducible manufacturing processes. BAdV-3 should consequently be regarded as a complementary veterinary vaccine-vector platform whose eventual practical value will depend on target-species efficacy and scalable product development.

## Figures and Tables

**Table 1:** Key milestones in adenovirus-based vector development relevant to BAdV vaccine platforms.

Period/Study Focus	Major Development	Relevance to BAdV-Based Vectors	Ref.
Early adenovirus discovery	Adenoviruses identified and characterized as non-enveloped, double-stranded DNA viruses	Established biological foundation for later vector development	[1,3]
Development of producer cells	HEK293 cells enabled production of E1-deleted replication-defective adenoviral vectors	Provided the conceptual basis for complementing cell lines used in BAdV vector production	[7,19]
First-generation adenoviral vectors	E1-deleted and E1/E3-deleted vectors developed for gene transfer	Influenced E1- and E3-based engineering strategies in BAdV-3	[2,3]
Gene therapy applications	Adenoviral vectors used for efficient transient gene delivery	Demonstrated high transgene expression but also strong immunogenicity	[8,9]
Transition to vaccine vectors	Immunogenicity became advantageous for vaccine antigen delivery	Supported use of adenoviruses for humoral and cellular immune induction	[3,5]
Alternative adenoviral platforms	Rare human and non-HAdV developed to reduce anti-HAdV-5 immunity	Provided rationale for BAdV as a non-human adenoviral vector	[15,16]
BAdV-3 vector development	BAdV-3 engineered to express foreign genes and vaccine antigens	Established BAdV-3 as a veterinary vaccine vector candidate	[17,18,19,20]

**Table 2:** Biological and engineering features of BAdV-3 relevant to viral vector design.

Feature	Description	Relevance to Vector Development	Ref.
Viral family	Member of *Adenoviridae*	Provides established adenoviral biology for vector design	[26,27]
Genome type	Linear double-stranded DNA genome	Allows genetic manipulation and insertion of expression cassettes	[32]
E1 region	Essential for viral replication	E1 deletion generates replication-defective vectors that limit productive replication in normal host cells and may therefore provide an improved biosafety profile; however, residual infectivity, recombination, biodistribution, and shedding must still be evaluated.	[19]
E3 region	Generally dispensable for replication in cell culture	Common site for transgene insertion	[17,18]
Capsid proteins	Hexon, penton base, fiber, pIX, and other structural proteins	Influence antigenicity, tropism, virion assembly, and vector rescue	[33,34]
Packaging motifs	Required for genome encapsidation	Must be preserved for stable vector rescue	[35]
Complementing cells	Producer cells supply deleted viral functions in trans	Required for replication-defective BAdV production	[19]
Mucosal tropism potential	Adenoviral vectors can transduce mucosal cells	Supports intranasal or mucosal vaccination strategies	[21,24]

**Table 3:** Experimental evidence for BAdV-based vector development and vaccine applications across in vitro, non-target animal, and target-species studies.

Evidence Level	Vector Design	Antigen/ Transgene	Model/ Species	Dose, Route, and Regimen	Immune Response/ Vector Outcome	Protection	Safety/Relevant Limitations	Ref.
Vector development/in vitro	Recombinant BAdV-3 with an expression cassette inserted in the E3 region	Firefly luciferase	Cell culture/vector-development system	Not applicable	Demonstrated that BAdV-3 could accommodate and express a heterologous transgene	Not applicable	Foundational vector-development study; no animal vaccination, protective-efficacy, or target-species safety assessment	[17]
Vector development/in vitro	E3-deleted recombinant BAdV-3 expressing full-length or truncated BHV-1 gD	Full-length or truncated BHV-1 gD	Cell culture/vector-construction system	Not applicable	Demonstrated construction and expression of vaccine-relevant BHV-1 gD antigens from E3-modified BAdV-3	Not assessed	Vector-construction and characterization study; no direct target-species protection or field-safety assessment	[18]
Vector development/in vitro	E1A-deleted replication-defective BAdV-3 produced using complementing cells	Recombinant expression-vector platform	Complementing producer cells/cell culture	Not applicable	Established rescue and propagation of replication-defective BAdV-3 and demonstrated feasibility of controlled heterologous gene expression	Not applicable	Productive replication is restricted in non-complementing cells; nevertheless, residual infectivity, recombination, genetic stability, and other vector-associated risks require evaluation during subsequent development	[19]
Target species	Replication-competent and replication-defective BAdV-3 expressing BHV-1 gD	BHV-1 gD	Calves	1 × 10^8^ PFU; intratracheal for the first and second immunizations, followed by subcutaneous administration for the third immunization	gD-specific systemic responses; replication-competent vector also induced stronger serum and nasal antibody responses	Partial protection with the replication-competent vector; replication-defective vector mainly primed gD-specific responses	Direct target-species evidence, but protection was partial; additional studies are required to define safety, duration of immunity, shedding, and field efficacy	[20]
Non-target model	BAd-H5HA	H5N1 HA	BALB/c mice	1 × 10^8^ PFU, IM, two doses 3 weeks apart	Humoral and cellular responses maintained despite high pre-existing HAdV-5 immunity	Complete protection against lethal H5N1 challenge	Non-target mouse model; efficacy and safety cannot be extrapolated directly to livestock	[22]
Non-target model	BAd-H5HA	H5N1 HA	BALB/c mice	1 × 10^6^–1 × 10^8^ PFU, single IN or IM dose	Dose- and route-dependent humoral and cellular responses	Complete protection at 1 × 10^6^ PFU IN and 3 × 10^7^ PFU IM	Dose-sparing effect was demonstrated in mice and was route dependent; livestock dose and safety require independent determination	[23]
Non-target model	BAdv85C5	Mycobacterial Ag85B-p25	C57BL/6 mice	1 × 10^7^ PFU, IN, single dose or booster after BCG vaccination	Strong pulmonary T-cell and memory responses	Reduced *M. tuberculosis* burden after aerosol challenge	Non-target mouse model; mucosal efficacy, shedding, local safety, and dose remain to be established in target livestock species	[24]
Non-target model	BAd universal influenza constructs	Influenza A/B NP-based antigens	BALB/c mice and ferrets	Mice: 5 × 10^7^ PFU IN once; ferrets: 5 × 10^7^ PFU IN twice, 3 weeks apart	Broad systemic, mucosal, and cellular immune responses	Protection against influenza A and B virus challenges in experimental models	Evidence derives from mice and ferrets; target-species veterinary efficacy and safety have not been established	[25]

**Table 4:** Advantages and limitations of BAdV-based viral vectors.

Aspect	Advantages	Current Limitations
Vector origin	Non-human adenoviral backbone may reduce interference from anti-HAdV immunity	Anti-BAdV immunity in cattle populations remains insufficiently characterized
Veterinary relevance	Bovine-origin platform is relevant to cattle vaccine development	Target-species efficacy data remain limited
Immunogenicity	Can induce humoral, cellular, and mucosal responses	Immune correlates of protection are not yet standardized
Mucosal vaccination	Potential for intranasal delivery against respiratory pathogens	Shedding, local safety, and mucosal dose optimization require further study
Genetic engineering	E1 and E3 regions allow rational vector design	Large or multivalent inserts may affect stability and yield
Safety	Replication-defective design limits productive replication and may improve the biosafety profile	Residual infectivity, biodistribution, shedding, recombination, and replication-competent contaminants must still be evaluated
Manufacturing	Complementing cell lines allow production of replication-defective vectors	Producer cell efficiency, scale-up, and quality control need optimization
Translation	Potential for BRDC, multivalent vaccines, and One Health applications	No widely used commercial BAdV-vector vaccine is currently established

**Table 5:** Comparative perspective of BAdV-3 and representative veterinary viral-vector platforms.

Vector Platform	Principal Veterinary Host	Major Strengths	Major Limitations	Position Relative to BAdV-3
BAdV-3	Cattle	Bovine origin; E1/E3 engineering; replication-defective option; respiratory/mucosal potential	Limited cattle efficacy, vector-immunity, shedding and manufacturing data	Emerging cattle-specific platform
HVT vector	Poultry	Early-life vaccination; prolonged antigen exposure; strong poultry efficacy; relatively resistant to some maternal-antibody interference	Host restricted; persistent herpesvirus biology; insert-dependent performance	Much more mature veterinary platform
Fowlpox vector	Poultry	Large recombinant capacity; long-lasting immunity; DIVA-compatible designs	Parenteral/wing-web delivery often required; anti-fowlpox immunity may interfere	More established but less suited to respiratory mass delivery
NDV vector	Poultry	Respiratory delivery; spray/mass vaccination; bivalent immunity	Vector/MDA interference; antigenic matching and insert stability important	Stronger mass-delivery experience
Capripoxvirus vector	Cattle, sheep, goats	Ruminant host relevance; multivalent potential; strong target-species protection demonstrated	Live-vector biosafety and vector-immunity considerations	Strong ruminant comparator with more direct challenge evidence
PRV vector	Swine	Large-genome host-adapted platform; bivalent/multivalent potential	Herpesvirus persistence and vector-immunity considerations	Stronger target-species multivalent proof of concept

**Table 6:** Comparative biological, engineering, production, and mucosal characteristics of BAdV-3 and representative veterinary viral-vector platforms.

Platform	Genome Size/Type	Insert Capacity	Replication Status	Seroprevalence/ Vector Immunity	Representative Production	Mucosal Performance	Ref.
BAdV-3	34,446 bp; linear dsDNA	~4.5 kb for E3/E4-deleted replication-competent vectors; ~5.0 kb for E1A/E3/E4-deleted replication-defective vectors	Both replication-competent and replication-defective designs available	Natural anti-BAdV-3 immunity occurs in cattle; one feedlot cohort had 17.8% seropositivity at entry and 54.8% seroconversion within 56 days	WT/E3-deleted vectors reached ~10^9^ PFU/mL; early E1A-deleted vectors ~10^6^–10^7^ PFU/mL; optimized E1-complementing cells supported ~1–2 × 10^8^ PFU/mL	Intranasal vaccination of calves induced serum and nasal antibodies and partial protection	[19,32,48,52,53,54]
HVT	159,160 bp; linear dsDNA herpesvirus genome	No single validated maximum; stable multigene and triple-insert recombinant HVT vectors demonstrated	Replication-competent, persistent, and predominantly cell-associated	Maternal/vector immunity is common in commercial poultry, but HVT-vectored vaccines may retain efficacy in the presence of maternal antibodies	Cell-associated production in avian cells; approximately 2000 viral genomes per infected cell reported in one experimental system; not directly comparable with volumetric infectious titers	Predominantly administered in ovo or subcutaneously; respiratory mucosal delivery is not a principal feature of the platform	[55,56,57,58,59]
Fowlpox virus	~288 kbp; linear dsDNA	Large multigene capacity; recombinant constructs with inserts at up to three genomic sites have been demonstrated	Replication-competent in avian cells; infection is generally abortive in non-avian cells	Previous fowlpox vaccination can reduce the performance of recombinant fowlpox-vectored vaccines	Production is well established in avian/chick-cell systems, although directly comparable volumetric infectious yields are inconsistently reported	Relatively weak by some mucosal routes; nasal and drinking-water administration were inferior to wing-web vaccination in an H5N2 model, with eyedrop administration also providing limited protection	[60,61,62,63]
NDV	~15.2 kb; nonsegmented negative-sense ssRNA	Usually accommodates an additional transcription unit; foreign-gene insertion may affect replication/yield; an experimental two-segment system increased coding capacity by ~30%	Replication-competent attenuated/lentogenic vectors commonly used	Anti-NDV immunity is common in vaccinated poultry; maternally derived NDV antibodies can interfere with rNDV-vectored vaccination	Recombinant NDV has reached up to ~10^9.8^ EID_50_/mL in embryonated eggs; yield can vary with construct and insert	Respiratory/spray delivery has been demonstrated in poultry and represents a practical option for mass vaccination	[64,65,66,67]
Capripoxvirus	~150 kb; linear dsDNA	Large genome; foreign-gene insertion into nonessential loci such as the TK locus has been demonstrated; a fixed maximum insert capacity has not been established	Usually live replication-competent attenuated vectors	Strongly dependent on endemicity and previous vaccination; pre-existing capripoxvirus immunity can influence recombinant-vector efficacy	Serum-free Vero/BelloStage production of recombinant SPPV reached mean titers of ~7.08–7.50 log_10_ TCID_50_/mL, substantially higher than static culture in one study	Most experimental vaccination has been parenteral; dedicated respiratory mucosal performance remains poorly characterized	[68,69,70,71]
PRV	143,461 bp; linear dsDNA	Large genome with multiple nonessential loci suitable for transgene insertion; a universal maximum insert capacity has not been defined	Live replication-competent attenuated or gene-deleted vectors predominate	Depends strongly on regional infection/vaccination status; maternally derived antibodies can interfere with active vaccine responses	A TK/gE-deleted vaccine candidate reached ~10^7.5^ TCID_50_/mL in ST cells; production is strain- and cell-system dependent	Intranasal administration is feasible, but latency/reactivation and vector shedding remain important biosafety considerations	[72,73,74,75,76]

## Data Availability

No new data were created or analyzed in this study. Data sharing is not applicable to this article.

## Abbreviations

The following abbreviations are used in this manuscript:

Ad26	adenovirus type 26
Ad35	adenovirus type 35
APC	antigen-presenting cell
BAdV	bovine adenovirus
BAdV-3	bovine adenovirus 3
BHV-1	bovine herpesvirus 1
BPIV-3	bovine parainfluenza virus type 3
BRDC	bovine respiratory disease complex
BRSV	bovine respiratory syncytial virus
BVDV	bovine viral diarrhea virus
CAdV	canine adenovirus
CAM	chorioallantoic membrane
CD4	cluster of differentiation 4
CD8	cluster of differentiation 8
CEF	chicken embryo fibroblast
ChAd	chimpanzee adenovirus
COVID-19	coronavirus disease 2019
DIVA	differentiation of infected from vaccinated animals
dsDNA	double-stranded DNA
E1	early region 1
E1A	early region 1A
E3	early region 3
E4	early region 4
EID50	50% egg infectious dose
gD	glycoprotein D
gG	glycoprotein G
H5N1	influenza A virus subtype H5N1
HAdV	human adenovirus
HAdV-5	human adenovirus type 5
HEK293	human embryonic kidney 293 cells
HIV	human immunodeficiency virus
HVT	herpesvirus of turkey
ICTV	International Committee on Taxonomy of Viruses
IFN-γ	interferon gamma
IgA	immunoglobulin A
IgG	immunoglobulin G
IM	intramuscular
ITR	inverted terminal repeat
MDA	maternally derived antibodies
MHC	major histocompatibility complex
MMP-9	matrix metalloproteinase 9
NDV	Newcastle disease virus
PAdV	porcine adenovirus
PFU	plaque-forming units
pIX	adenovirus protein IX
Poly(A)	polyadenylation signal
PRV	pseudorabies virus
RBMX2	RNA-binding motif protein X-linked 2
rNDV	recombinant Newcastle disease virus
SARS-CoV-2	severe acute respiratory syndrome coronavirus 2
SC	subcutaneous
SPPV	sheeppox virus
ssRNA	single-stranded RNA
TCID50	50% tissue culture infectious dose
TK	thymidine kinase
WT	wild type
